# Contestations and complexities of nurses’ participation in policy-making in South Africa

**DOI:** 10.3402/gha.v7.25327

**Published:** 2014-12-22

**Authors:** Prudence Ditlopo, Duane Blaauw, Loveday Penn-Kekana, Laetitia C. Rispel

**Affiliations:** Centre for Health Policy & Medical Research Council Health Policy Research Group, School of Public Health, Faculty of Health Sciences, University of the Witwatersrand, Johannesburg, South Africa

**Keywords:** nurses, participation, policy-making, contestations, South Africa

## Abstract

**Background:**

There has been increased emphasis globally on nurses’ involvement in health policy and systems development. However, there has been limited scholarly attention on nurses’ participation in policy-making in South Africa.

**Objective:**

This paper analyses the dynamics, strengths, and weaknesses of nurses’ participation in four national health workforce policies: the 2008 Nursing Strategy, revision of the Scope of Practice for nurses, the new Framework for Nursing Qualifications, and the Occupation-Specific Dispensation (OSD) remuneration policy.

**Design:**

Using a policy analysis framework, we conducted in-depth interviews with 28 key informants and 73 frontline nurses in four South African provinces. Thematic content analysis was done using the Atlas.ti software.

**Results:**

The study found that nurses’ participation in policy-making is both contested and complex. The contestation relates to the extent and nature of nurses’ participation in nursing policies. There was a disjuncture between nursing leadership and frontline nurses in their levels of awareness of the four policies. The latter group was generally unaware of these policies with the exception of the OSD remuneration policy as it affected them directly. There was also limited consensus on which nursing group legitimately represented nursing issues in the policy arena. Shifting power relationships influenced who participated, how the participation happened, and the degree to which nurses’ views and inputs were considered and incorporated.

**Conclusions:**

The South African health system presents major opportunities for nurses to influence and direct policies that affect them. This will require a combination of proactive leadership, health policy capacity and skills development among nurses, and strong support from the national nursing association.

Notwithstanding the extraordinary and influential roles of many nurse leaders in the development of patient care and population health policies (1–3), several studies have highlighted the need for increased nurses’ involvement and participation in policy processes (4–12). ‘Participation’ in this context refers to actions and procedures designed to inform, consult, and involve the ‘community of nurses’, including nurse leaders and frontline nurses, to allow them to make inputs into those decisions that affect them (13). The International Council of Nurses (ICN) has stressed the importance of nurses’ contribution to health services planning and their participation in policy development (14). Considerable progress has been made in advancing nurses’ presence, role, and influence in health policy development (1, 15), illustrated by an increasing number of nurses elected as political office bearers and/or appointed to national and international boards (1).

In high-income countries such as Australia, Canada, the United Kingdom, and the United States of America, professional nursing associations are important platforms for individual nurses and the nursing profession to exercise power and influence policy (16–18). A recent review found that the common policy issues addressed by nursing associations include broader population health issues as well as professional or practice issues such as the scope of nursing practice, prescribing rights, education requirements, and workplace issues such as nursing shortages (19). There are examples from low-and middle-income countries (LMICs) where nurses have worked either individually or collectively through professional organisations to advocate for enabling health policies (20). In Rwanda, the chief nurse and the national nursing association mobilised support for legislation to improve the quality of nursing education and professional standards (20). In Paraguay, the nursing association capitalised on the visit of the chief executive officer (CEO) of the ICN to highlight poor staffing levels in health facilities in the country and to propose solutions to address them (20). In Kenya, the Nursing Council was a critical stakeholder in the development of the national electronic database on the nursing workforce (21).

Despite these encouraging developments, Leavitt (10) has argued that nurses are largely absent in health policy reforms compared to other professional and health interest groups. Their sub-optimal participation and limited role in health policy decisions is more acute in LMICs (10, 22, 23), where recent studies have underscored the need for increased nurses’ involvement in broader political and health policy debates (6, 8, 12), specifically with regard to HIV and AIDS policies (4, 11, 22). These LMIC participation studies have used different research designs, methods, and participants, including: a descriptive, mixed method study of hospital-based professional nurses and nurse leaders in Thailand (9); a qualitative case study with frontline nurses, nurse managers, and decision-makers in Kenya (4); in-depth interviews with policy-makers and a small survey with registered nurses in Botswana (22); a quantitative, descriptive study with registered nurses in Nigeria (6); and a Delphi-survey with nurse leaders from three East African countries (12). These studies have found that nurses have high levels of knowledge about national health policy development (9) on HIV and AIDS policies (22), but their role and participation in these policies were limited (4, 6, 9, 11, 12, 22). In those instances where nurses were involved, participation was limited to the policy implementation phase, rather than the full policy cycle from development to monitoring and evaluation (9, 12).

Phaladze (22) found that policy-makers were of the opinion that nurses did not have the expertise to participate in policy decisions, supporting the finding from other studies that the lack of policy and political skills is a hindrance for nurses’ policy participation (4, 9). Other barriers to nurses’ participation included: limited skills in public relations affecting their ability to explain and promote nursing (9); competing priorities (4, 23); insufficient time (4, 24); lack of resources (11, 24); insufficient involvement in policy formulation committees (9); and sub-optimal communication (11).

In South Africa, the Ministry of Health has emphasised the critical role of nurses in the implementation and success of health sector reforms towards universal health coverage (25). However, with the exception of a study that focused on the role of nurses in AIDS policy development (11), we could not find studies that explore nurses’ participation in broader health workforce policies. In light of limited empirical evidence and the centrality of the health workforce, specifically nurses, to health sector reforms (26, 27), this paper analyses the dynamics, strengths, and weaknesses of nurses’ participation in four national policies: the 2008 Nursing Strategy, revision of the Scope of Practice for nurses, the new Framework for Nursing Qualifications, and the Occupation-Specific Dispensation (OSD) financial incentive policy.

## Methods

The study was approved by the University of the Witwatersrand Human Research Ethics Committee (Medical) and the Provincial Health Research Ethics Committees in the four participating provinces (Eastern Cape, Free State, Gauteng and Western Cape). Hospital managers also provided permission to access their facilities. All participants received a study information sheet and gave informed written consent.

### Study design

A multiple descriptive case study design, informed by the Walt and Gilson policy analysis framework (28), was used. The framework focuses on four related factors critical to understanding public policy-making: actors, policy content, contextual factors, and process (28).

### Policies of interest

The study focused on four national policies: the 2008 Nursing Strategy, the Scope of Practice for nurses, the Framework for Nursing Qualifications, and the OSD.

The 2008 Nursing Strategy aimed to address the national nursing crisis by proposing action in six key strategic areas: nursing practice, nursing education and training, nursing leadership, nursing regulation, social positioning of nursing, and resources for nursing (29). The revised Scope of Practice for nurses is a legal document that outlines the role, responsibilities, and functions of different categories of nurses in the health system, whereas the new Framework for Nursing Qualifications is concerned with aligning nursing qualifications with the National Qualifications Framework. The OSD is a financial incentive strategy intended to attract, motivate, and retain health professionals in the public sector (30).

The selection of these four policies was influenced by two factors: prioritisation by key nursing stakeholders at a consultative workshop held in 2008 (31); and the policies represented different stages of policy development. For instance, the revisions of the Scope of Practice and the Qualifications Framework were at the development stage, the Nursing Strategy was at the initial phases of implementation and the OSD was fully implemented. These policies also differed in focus, the main policy drivers and actors involved, the degree of contestation about content, and the nature of the participatory processes.

### Study sites and setting

The study was conducted between 2009 and 2011 in four provinces in South Africa: Gauteng, Eastern Cape, Free State, and the Western Cape. The selected provinces were already part of a broader multi-year programme of research on nursing, initially chosen to allow for geographical comparisons (urban–rural) and possible variations in the interpretation and implementation of the selected policies. In each province, we randomly selected one hospital from each of the clusters of academic, regional, district, and specialised hospitals. The final sample consisted of 16 hospitals, four in each province.

### Study participants and data collection

Using a snowballing sampling technique, 28 key informants were selected purposively on the basis of their knowledge, involvement, or influence with the policy-making processes of the policies of interest. The key informants comprised the following groups of stakeholders: national government (*n*=6), provincial government (*n*=8), private sector (*n*=1), nursing educators/academics (*n*=7), statutory body (*n*=1), nursing association (*n*=4), and international non-profit global health organisation (*n*=1). The selected key informants were interviewed using a pilot-tested, semi-structured interview guide focusing on: the extent and nature of nurses’ participation and involvement in policy-making (both in general and in relation to the specific policies); the roles, interests, and influence of different nursing actors on these policies; and recommendations for improving nurses’ participation and involvement.

Semi-structured interviews were also conducted with 73 frontline nurses in the 16 selected hospitals, consisting of operational managers (*n*=15), professional nurses (*n*=15), enrolled nurses (*n*=13), enrolled nursing assistants (*n*=16), and shop stewards (*n*=14). The questions focused on the respondents’ awareness and knowledge of the policies under investigation, perceptions about the extent and nature of frontline nurses’ participation and involvement in nursing policy-making, and recommendations for improving nurses’ participation and involvement.

### Data analysis

A thematic content analysis of transcripts was conducted (32) using the Atlas.ti software. Once transcripts were loaded into the software, a line-by-line coding of each transcript was done using both inductive and deductive approaches to identify recurring themes. Thereafter, axial coding was conducted to identify connections and linkages in codes based on the conceptual framework (28). These themes and codes were used to organise the results in this paper. Three researchers (PD, DB, LPK), who received formal training in Atlas.ti, independently read and coded at least 12 transcripts and discussed discrepancies until agreement on the codes was reached, thereby ensuring coding consistency. To ensure the trustworthiness of the data, continuous peer debriefing and checking of researchers’ interpretations against the raw data was done. Where necessary, codes were renamed and redefined.

## Results

Three broad themes emerged from the analysis: the extent of nurses’ participation in nursing policies, the nature of participation, and contestations and complexities of participation.

### The extent of nurses’ participation in nursing policy processes

There was recognition and appreciation amongst several key informants that democracy created numerous opportunities for nurses’ participation in policy development. This was in contrast to the imposition of policies under apartheid. This increased ‘policy space’ occurred within the context of South Africa's rights-based Constitution. One key informant noted that:

Since 1994, there has been a lot more discussion and a lot more participation [of nurses]. Pre-1994, we were just sort of told ‘here is a policy’. (KII 7, Gauteng Department of Health)

When asked about nurses’ participation in the development of the four policies of interest, all key informants pointed to the wide range of policy actors involved to a greater or lesser degree in the development of these policies. However, there were contradictory views on participation, particularly in the case of the 2008 Nursing Strategy. Key informants from the National and Provincial Departments of Health were of the opinion that the process was nurse-led and that nursing leaders and representatives from all sections of the nursing profession had been involved, as can be seen from the excerpt below:

[The] Nursing Strategy is driven by nurses themselves because it started nationally when nurse leaders were called in to start with the Strategy. (KII 11, Eastern Cape Provincial Department of Health)

This was in contrast to respondents from outside the National and Provincial Departments who were of the opinion that the process and development of the Nursing Strategy were led by staff in the National Department who were not nurses. One said:

Very few nurses were involved in the development of the Nursing Strategy. Nurses were not at the policy table. (KII 26, Nursing Academic)

There was also a strong sense amongst frontline nurses and several key informants that frontline nurses were excluded from participation in broader health workforce policies. Frontline nurses believed they were excluded from policy processes because of the failure of policy-makers to recognise the importance of their clinical knowledge and expertise in informing policies. Some of these nurses commented that:

I don't understand how they [policy-makers] make decisions about nurses without involving the nurses. (Shop steward 37, Regional Hospital, Eastern Cape Province)

There are things that are decided by National [the National Department of Health] but when you are in the ward you see things practically and when you compare it to the policies, these [policies] are unrealistic. (Professional nurse 24, Regional Hospital, Gauteng Province)

Although respondents acknowledged that the participation of frontline nurses is important, some informants were of the view that it is impractical to involve all the nurses considering that they are the largest group of health professionals.

I think that in policy development, it would be impossible to get all the 120 or 140,000 nurses into one room and say ‘do you agree with this policy that has been proposed’. (KII 39, National Department of Health)

**Fig. 1:**
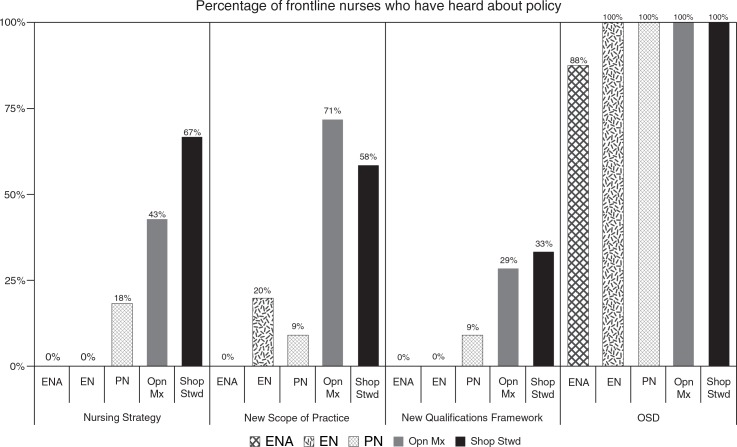
Frontline nurses awareness of nursing policies. ENA: enrolled nursing assistant; EN: enrolled nurse; PN: professional nurse; Opn Mx: operational manager; Shop Stwd: shop steward.

The study found that the nature of the policy determined the level of awareness of frontline nurses. Figure 1 shows nurses’ levels of awareness of the four policies analysed in this study.

One hundred percent of enrolled nurses, professional nurses, operational managers, and shop stewards, and 88% of enrolled nursing assistants, reported that they have heard about the OSD policy. This was in contrast to the other three policies, where the level of awareness on policies was much lower. Operational managers and shop stewards were more likely to know about policies than other categories of nurses. The level of awareness among operational managers ranged from a low of 29% for the new Qualifications Framework to 71% for the new Scope of Practice for nurses. In the case of shop stewards, the level of awareness ranged from a low of 33% for the new Qualifications Framework to 67% for the Nursing Strategy. Figure 1 also shows that the level of awareness on the three policies among the different categories of general nurses was very low. One of the reasons mentioned was the lack of feedback by managers who attended the meetings where these policies were discussed.

### The nature of nurses’ participation

The majority of study participants were of the opinion that there was insufficient involvement of nurses in policy development. One key informant, reflecting on the OSD policy processes, commented that even though there was consultation with nursing leaders, their views were not incorporated into the policy, thus leading to unintended consequences during policy implementation.

It was a very top down approach such that those of us who were academics from the universities, I remember we actually left that meeting feeling like ‘what's the point’? There has always been a doctor behind it and telling nurses in a very top down way of saying ‘this is what the DoH [Department of Health] has decided and you shall do that!’ (KII 17, Nursing Academic)

A number of key informants were also of the view that nurse leaders are reactive rather than proactive when it comes to nursing policy processes. With regard to the Scope of Practice and Qualifications Framework policies, one key informant commented that nurse leaders often participate in policy-making when decisions have already been concluded:

When I think of nurse leaders, I think we are reactive because policies are being made in different areas, be it health, be it education, and we always seem like we come at a point when it is done and dusted; that's where we always find ourselves most of the time. We are happy to respond to what people say about us rather than challenge them. (KII 8, Gauteng Department of Health)

Another key informant was of the view that even though some nurses hold senior positions in provincial government and have the potential to influence policy, these nurses are not assertive enough to ensure that changes that could affect the nursing profession are achieved:

The challenge in South Africa about nursing is that, while we have nurses that are holding key positions, those nurses sometimes don't even have ‘teeth’ to change whatever they want to change. (KII 3, Nursing Association)

The key informants’ interviews thus identified two main categories for describing the nature of nurses’ participation in the policy processes. Individual participation happened when participants felt that their invitation to participate and be involved was on an individual basis and not intended to represent any specific interest group in nursing. Collective or representational participation happened when participants viewed their invitation to participate and be involved as representing a specific nursing interest group, such as the national nursing association, educators, or academia. Hence, participants’ views on the level and nature of participation differed depending on the policy of interest. For example, with regard to the Scope of Practice, the Qualifications Framework, and the OSD, the key actors were of the opinion that they either represented a particular nursing structure or interest group. With regard to the development of the Nursing Strategy, some informants were consulted in an individual capacity:

I made comments as an individual …. I participated as a consultant (KII 32, Nursing Academic)

I was involved and worked in a province but I wasn't representing a province – just sitting as an individual. (KII 7, Gauteng Department of Health)

Hence these informants appeared to view individual participation in the Nursing Strategy as a relatively minor contribution as it did not represent the views of the majority of nurses. According to key informants, the general approach to involving nurses in some of these policies tended to be formal and included activities such as serving on committees or boards, providing inputs into draft policy documents, chairing committees and/or trade union representation in collective bargaining processes. Because of the small pool of nurse leaders to draw from, the tendency of policy-makers to focus on multiple nursing policy issues in a short space of time required those involved to shift their attention constantly from one policy to another. They indicated that the OSD was introduced in 2007 but was implemented in 2008, whereas the Nursing Strategy was introduced in 2008. Around the same time period, debates around the Scope of Practice and the Qualifications Framework were also happening. One informant commented:

And I was involved in the Nursing Strategy after that, I was swallowed up in the OSD stories. Most of my time went into the OSD. (KII 8, Gauteng Department of Health)

With regard to the non-participation of frontline nurses, some informants were of the view that in a few instances where platforms were created to involve these nurses – such as inviting them to workshops – they seldom participated because they were intimidated by the presence of their managers. Overall, some of the reasons provided by key informants for the perceived limited participation of the broader nursing community in nursing policy processes include: the dominance of physicians and ‘others’ such as hospital CEOs and human resource managers, the under-representation of nurses in leadership positions, the predominantly female nature of the profession, and lack of training in policy and networking skills.

The other issue that I really see as a problem is that nurses as a whole are not trained in policy, they don't understand what policy is about and they don't understand the policy cycle. If you don't understand the policy cycle, then you will not know when there is a window of opportunity to influence policy. (KII 26, Nursing Academic)

We need to learn to be politically clever. Nurses don't have the skills of networking like businessmen do at the golf course. (KII 3, Nursing Association)

In general, a number of key informants commented that nurses themselves are to blame for their limited involvement and participation in policy-making processes because they tend to adopt a passive role in the policy making process. This is mainly indicated through statements such as ‘nurses are not given an opportunity’ to participate in nursing policy processes or that ‘policies are developed without us’. Interestingly, some informants were optimistic that if nurses changed their way of thinking, they could turn the situation around and achieve maximum participation in health policy processes. Some informants commented that:

Unless the profession claims back what is ours, people shouldn't have this thing of, ‘you know we have not been consulted when the policy was being made?’ If you really stand your ground as the leader in your profession, and taking leadership in that regard, you will be involved in policy development. (KII 33, International Non-profit Health Organisation)

On the one hand, we are saying we are the backbone of the health care service, but we don't behave like we are the backbone. I think every nurse leader should make it her business to get involved in policy development. She should be staying close to debates and know exactly what is going on. (KII 25, Nursing Manager)

### Contestations and complexities of consultation and participation

The majority of key informants complained about the lack of cohesion or lack of collective action amongst different nursing stakeholders, which include the national nursing association, professional interest groups, university nursing academics, college nursing educators, nursing managers, the nursing council, and private sector nurses. These internal divisions discouraged collaboration among different nursing groups and made it difficult for nurses to have a unified voice, thus having a negative impact on how nurses were viewed by external actors, especially by key policy-makers. One informant explained:

We wanted to see the Minister of Health, but the Minister indicated that ‘Until the nurses have got one voice, I don't want to see them because sometimes there will be DENOSA [Democratic Nursing Organisation of South Africa] whose approach is more trade union kind of, there will be a group from the college, a group from the clinical side, a group from all those private sector people and you are all talking different things. Can you nurses just make up your mind and come as one voice to see me?’ (KII 32, Nursing Academic)

There were also apparent tensions expressed with regard to the position of nursing within the organisational structure of the National and Provincial Departments of Health. Depending on the province, the Nursing Division could report to Corporate Services, Hospital Services, or Human Resources for Health Directorates. A consistent view was that this hierarchy and lack of seniority of the designated nurses made it even more difficult to influence policy:

Most of the provinces, Nursing Directorates are reporting to the Hospital Service Managers who have a different mandate altogether because their interests is about hospitals, it's not about nurses only. (KII 3, Nursing Union)

In addition, some key informants also discussed the contestations around representation and the perceived legitimacy of some nursing groups. This was influenced by status and power relations within the profession. For example, there was a strong perception amongst some key informants that nurse academics or private sector nurses, had more prestige than those from nursing colleges or public health services. Therefore, there was a sense amongst these informants that prioritisation of nursing issues depended on how influential a certain group was and the agenda being pursued by a particular group. This tension was most prominent in relation to the Nursing Qualifications Framework.

Who is the Nursing Leadership because when I go there, they don't see me as representing a nursing voice? They would say ‘No, you are an academic’. (KII 32, Nursing Academic)

Several key informants expressed concerns about officials in the Human Resource Directorate of the National Department of Health who were leading the development of these four national policies, but who had limited or no understanding of the needs and expectations of nurses and the complexity of the nursing profession.

We keep on allowing other people to make decisions for us, people who don't understand how we work, people who don't understand how we are trained or how we think sometimes. (KII 27, Nurse Manager)

## Discussion

This study found that South Africa's democracy created opportunities and increased nurses’ participation in health policy development. Different nursing stakeholders – the national nursing association, professional interest groups, university nursing academics, college nursing educators, nursing managers, the nursing council, and private sector nurses – were involved to a greater or lesser degree in the development of the four national policies of interest.

However, the study found that nurses’ participation in policy-making is both complex and contested. There was a disjuncture between nursing leadership and frontline nurses in their levels of awareness of the four policies analysed. Frontline nurses were generally unaware of the 2008 Nursing Strategy, the revised Scope of Practice for nurses, and the new Framework for Nursing Qualifications. The exception was their awareness of the OSD remuneration policy as it affected them directly. However, even within this group of frontline nurses, there were different levels of awareness with operational managers and union shop stewards more likely to know about the four policies of interest, compared to other categories of nurses. A minority of frontline nurses were aware of the new Framework for Nursing Qualifications which will change nursing education radically when implemented. These findings were in contrast to studies in Thailand (9) and Botswana (22) where nurses had high levels of knowledge about the health policies under investigation. The reason could be because these studies focused on nursing leaders, rather than on frontline nurses.

The majority of key informants and frontline nurses were of the opinion that nurses’ participation in nursing policy development was sub-optimal, thus supporting the findings of other studies of limited nurses involvement in health policy development (4, 6, 9, 11, 12, 22). Our study revealed various contestations regarding the extent and nature of nurses’ participation in nursing policies. Although there was consensus of the importance of frontline nurses, and expressed discomfort about their exclusion from policy participation, study respondents also acknowledged the practical difficulties of involving thousands of frontline nurses in broader health policy development, and overcoming the barriers to their active participation in forums which include their managers. This lack of active participation of frontline nurses could be explained by their position in the health hierarchy, where junior nurses are expected to follow orders, rather than question their seniors within nursing (33, 34).

Shifting power relationships influenced who participated (individual or stakeholder group), with contestations regarding the legitimacy of the different nursing stakeholder groups. This is not surprising as Buse et al. (35) have pointed out that organisations or groups may not all speak with one voice because they are made up of many different people whose values and beliefs may differ. These authors also argued that the decision-making process in the policy arena depends on the policy issue, its significance, the political system within which the policy is being made, the power of the various actors, and reconciliation of the different views of the interest groups (35). Therefore, contestation during policy-making should rather be understood as reflecting the reality that the nursing profession is not uniform and that policy-making is a struggle between groups with competing interests.

The degree to which nurses’ views and inputs were considered and incorporated was also contested, with the OSD policy given as an example of the unintended consequences that occurred during policy implementation, because these perspectives or insights from nurses were ignored. These consequences included demoralisation of frontline nurses; and adverse relationships between managers and nurses, and among different categories of nurses (36, p. 141).

The sub-optimal involvement of nurses in health policy development was exacerbated by both internal and external barriers. Internal barriers to nurses’ participation or involvement in broader health policies included the perceived reactive (as opposed to a more proactive) approach of nursing leadership; their relative lack of assertiveness and notions of victim mentality, even when they held senior provincial government positions; the small number of nurses with policy and/or advocacy skills; and the lack of cohesion or lack of collective action amongst different nursing stakeholders. A major external barrier was the position of nursing within the health hierarchy and organisational structures in South Africa. For example, at the time when this study was conducted, there was no chief nursing officer (CNO) or nursing directorate in the National Department of Health, the human resource division was headed by a medical doctor, and there were no nurses dealing with any of the four policies that primarily affected nurses. Buse et al. have noted that doctors were often more influential in public health policy either as civil servants or as health ministers (35), while Shariff and Potgieter (12) also found that nurses were mostly invisible and that the health policy agenda in Kenya, Uganda, and Tanzania was dictated by other health professionals, notably doctors. Other studies have also found that the relative position and power of midwives (37) lack of supportive organisational structures (38), inadequate political and policy development skills (9), competing priorities (4, 23), insufficient time (4, 24), lack of resources (11, 24), insufficient involvement in policy formulation committees (9), and sub-optimal communication (11) combined to produce a complex set of factors that mitigate against maximum participation of the nursing profession in health policy development. In South Africa, barriers to nurses’ participation in turn are shaped by dynamics of race, class, and sex (33, 34). Nonetheless, it would be erroneous to conclude that South African nurses are powerless or without agency. Rather, as Webber has pointed out, ‘nurses need to be recognised as active players whose involvements are among those structuring, reinforcing, or resisting their current realities’ (39, p. 9).

This paper makes an important contribution to both the national and international literature on health policy analysis. However, the findings may not be generalisable as the study was limited to four South African provinces and key informants were selected purposively. The data gathered also represent the perceptions of key informants and frontline nurses at a point in time. Nonetheless, the study provides rich insights into the dynamics, strengths, and weaknesses of nurses’ participation in four national health workforce policies.

Since the study was conducted, the first CNO in democratic South Africa has been appointed. The appointment of the CNO is a positive development, as the experience in other countries has shown the impact of such an appointment on cohesion and collective action by the nursing profession (1, 14). In light of the study findings, we recommend three strategies to increase nurses’ participation and involvement in health policy development. First, the CNO should provide leadership and serve as the ‘glue’ that brings together different nursing stakeholders to discuss and implement the recommendations contained in the National Strategic Plan on nursing education, training, and practice (25). Second, the national nursing association should develop its own policy machinery to ensure that it has the capacity and skills to analyse, comment on, or lead the development of health policies. Third, the training of all nurses should include modules on health systems and policy development processes, leadership, and advocacy skills. Last, all work settings should explore simple, low-cost mechanisms to provide feedback on health sector or policy developments and to give nurses a voice to make inputs, in line with the recommendation at the 2013 Third Global Forum on Human Resources for Health (27).

## Conclusions

The importance of nurses to the success of health sector reforms in South Africa is unquestionable. There is evidence of the benefits to the health care system, patients, and the nursing profession when nurses are involved in health policy development (38, 40, 41). Nurses’ participation in the development of policies and strategies also enhances their job satisfaction and retention in the health sector (42). The South African health system presents major opportunities for nurses to influence and direct policies that affect them. This will require a combination of proactive leadership, health policy capacity and skills development among nurses, and strong support from the national nursing association.
